# Complete absence of the suprascapular notch in a Nigerian scapula: A possible cause of suprascapular nerve entrapment

**DOI:** 10.4103/0973-6042.44146

**Published:** 2008

**Authors:** David A. Ofusori, Raymond A. Ude, Christina U. Okwuonu, Olamide A. Adesanya

**Affiliations:** Department of Anatomy, School of Basic Medical Sciences, Igbinedion University, Okada, P.M.B 0006, Benin City, Edo State, Nigeria

The suprascapular notch is situated in the lateral part of the superior border of the scapula, just adjacent to the base of the coracoid process. This notch is converted into a foramen by the superior transverse scapular ligament and serves as a passage for the suprascapular nerve which supplies motor branches to the supraspinatus, infraspinatus, and sensory branches to the rotator cuff muscles, and the ligamentous structures of the shoulder and acromioclavicular joint. Rengachary *et al.*[1] have reported six different types of anatomical variations of the suprascapular notch. In some cases, the variation in the suprascapular notch is accompanied by a variation of the superior transverse scapular ligament.[2] These variations have a role to play in suprascapular nerve entrapment. Injury to the nerve may result in significant rotator cuff dysfunction.

**Figure 1:**
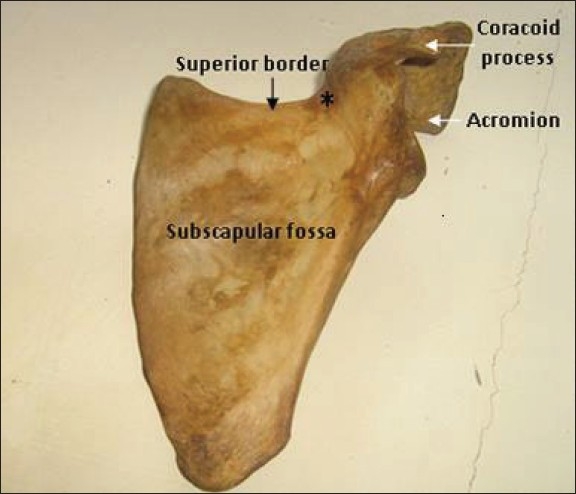
Costal surface of the entire length of the left scapula showing absent suprascapular notch. *: site for suprascapular notch if present.

**Figure 2:**
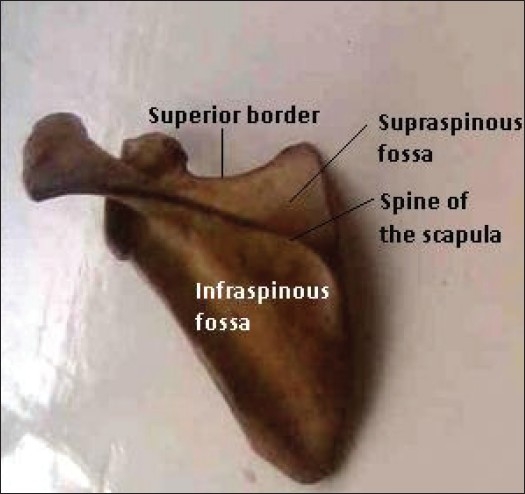
Posterior surface of the entire length of the left scapula showing absent suprascapular notch. Note its division into supraspinous and infraspinous fossae by the spine of the scapula.

Complete absence of the suprascapular notch has not been previously described amongst Nigerians. In this case that we report, the superior border presented a somewhat concave pattern at the mid region without any lateral confluent at the point where the suprascapular notch should have been; thus confirming its absence [Figures 1 and 2]. At the place where the suprascapular notch should have been present the bone was thinner and more translucent (indicated by the ^“*”^ in Figure 1) than in the other parts. This was different from what was seen in the normal right scapula, where the superior border of the scapula is relatively straight, with an indentation (suprascapular notch) at the junction of the medial two-thirds and the lateral third, just medial to the base of the coracoid process. In this paper, we describe a case of complete absence of the suprascapular notch in a Nigerian adult male scapula: which could be a possible cause of suprascapular nerve entrapment.

During routine bone extraction from cadavers for osteological study at the Department of Anatomy, Igbinedion University, Okada, Nigeria, we observed that the left scapula of a male cadaver presented a superior border without a suprascapular notch [Figures 1 and 2]. The length of the superior border, from the base of the coracoid process to the medial angle, was 6.5 cm; the length of the medial border, from the medial angle to the inferior angle, was 17.6 cm; while the lateral border, from the infraglenoid tubercle to the inferior angle of the scapula, was 14.2 cm. The weight of the entire left scapula was 79.3 g, the length was 15.2 cm, and the width was 10.2 cm and 5.8 cm, at the superior third and the inferior third, respectively.

The left scapula investigated in this study had prominent surface markings. The costal surface was concave, while the posterior part was divided by the spine of the scapula into supraspinous and infraspinous fossae for attachment of the supraspinatus and infraspinatus muscles, respectively, just as in normal cadaveric specimens [Figures 1 and 2].

The suprascapular notch is usually present in every scapula. It is commonly bridged by the superior transverse scapular ligament[4] (or bone in some cases[5]) and thus converted into a foramen, which is called the suprascapular foramen.

A thorough search of the literature revealed no report of absence of the suprascapular notch in a Nigerian scapula – this appears to be the first. Various factors have been identified as being causes of suprascapular nerve entrapment, including variation in the shape of the suprascapular notch[1,6] along with a thickened superior transverse scapular ligament.[7] It could be postulated that complete absence of the suprascapular notch may also be one of the predisposing factors for the suprascapular nerve entrapment syndrome. The suprascapular nerve is a motor nerve originating from the upper trunk of the brachial plexus (C5 and C6).[4,8] Suprascapular nerve entrapment may occur at any point along its course.[1] The absence of the suprascapular notch in our subject suggests the possibility of compression of the suprascapular nerve by the superior transverse scapular ligament on the superior border of the scapula. This compression may be pronounced when the superior transverse scapular ligament is ossified.[5] With entrapment of the nerve, atrophy of both the infraspinatus and supraspinatus muscles may occur. Black *et al.*[8] reported that paralysis, weakness, numbness, and burning sensations in the hand may be the initial symptoms; later, there may be only weakness of abduction and external rotation, as is seen in suprascapular injury.

The present investigation indicates that absence of the suprascapular notch can occur in the Nigerian population.
